# Functional Distinctions in Feeding Behavior of Two Herbivorous Reef Fish Lineages

**DOI:** 10.1093/iob/obag056

**Published:** 2026-09-21

**Authors:** Ava Luenenborg, Michalis Mihalitsis, Peter C Wainwright

**Affiliations:** Department of Evolution and Ecology, University of California, Davis, CA 95616, USA; Department of Ecology and Evolutionary Biology, University of California, Irvine, CA 92697, USA; Marine Laboratory, University of Guam, Mangilao, GU 96923, USA; Department of Evolution and Ecology, University of California, Davis, CA 95616, USA

## Abstract

Understanding the mechanisms and variation of how herbivorous fishes feed is key to understanding the dynamics of herbivory on coral reefs. In our comparative study of closely related nominally herbivorous fishes, we investigate the use of 10 distinct behaviors used to support feeding on algae. We found that species within Siganidae share 5 out of the 10 behaviors with Acanthuridae while also exhibiting two additional family-specific behaviors: presence of nibbling and extra jaw closure. Siganids predominantly use movements of the jaws whereas acanthurids also employ body and head movements while detaching algae from the substrate. A comparison among siganid species revealed an axis of diversity in the frequency and extent of their feeding behaviors, with the main axis of variation being strongly associated with sociality (schooling vs. pairing) and morphology (short-snouted vs. long-snouted). Overall, we show that siganids and acanthurids display distinct behaviors during feeding. Thus, our results suggest potential niche differences between 2 major groups of herbivorous reef fishes.

## Introduction

Coral reefs are among the most biodiverse ecosystems on the planet. A large part of this diversity is made up of reef associated fishes, which are successful in utilizing the diverse variety of food resources on reefs. To acquire these resources, reef fishes have evolved diverse feeding mechanisms, including directly biting prey that require detachment (Peoples et al. 2026). Species that directly bite their prey are estimated to make up 40% of modern reef fish species (Corn et al. 2022). Perhaps the most widely recognized and studied group of biters are herbivorous fishes, which remove turf algae, macroalgae, cyanobacteria, and detritus from reefs (Horn 1989; Choat et al. 2002; Wilson et al. 2003; Clements et al. 2016; Duran et al. 2019). As coral reefs are becoming increasingly threatened due to anthropogenic stressors (Hoegh-Guldberg 2011; Wernberg et al. 2024), these fishes are especially important to reef resilience, because their grazing activity helps prevent algal overgrowth (Bellwood et al. 2006; Burkepile and Hay 2008). Turf algae specifically is a major benthic resource on reefs and is becoming increasingly dominant on degraded reefs (Tebbett et al. 2023). Understanding how herbivorous reef fishes remove turf algae is therefore important in understanding coral reef resilience.

Herbivory has evolved independently in several reef fish lineages that exhibit different feeding morphology and behavior (Bellwood et al. 2014). Studies that document feeding behaviors at the bite level (e.g., jaw, body, and fin motions) using high-speed videography can clarify how different fish remove their target prey, and thus, provide insights on functional distinctions among herbivores. Several studies have focused on surgeonfish (Acanthuridae) which show substantial diversity in how different species detach algae (Purcell and Bellwood 1993; Perevolotsky et al. 2020; Mihalitsis and Wainwright 2024; Mihalitsis et al. 2025a; Mihalitsis et al. 2025b). High-speed filming of acanthurids has identified common behaviors used during feeding that aide in removing algae from the substrate. However, there are other ecologically important groups of herbivorous reef-associated lineages for which there is little to no evidence on how they detach algae from reefs.

One such understudied group are the rabbitfishes (Siganidae), named for their characteristic rabbit-like “nibble” when feeding. Of the 31 species, five possess elongate snouts, a morphological trait also evolved independently in acanthurids that has been shown to have important ecological relevance in both families (Brandl and Bellwood 2014; Brandl and Bellwood 2015; Nanami 2018). Siganids and acanthurids show several morphological similarities that appear to be convergently evolved, including a laterally compressed deep-body shape, and short mandibles with a single row of often multicusped teeth that sit upright in the jaws (Tyler and Bannikov 1997; Huertas and Hemingson 2025) rather than being directed anteriorly as in the acanthurid sister group *Zanclus* (Mihalitsis et al. 2025a). Functional groups have been identified among siganids based on how and what they feed on, and include browsers feeding mainly on leathery macroalgae, croppers feeding mainly on red and green macroalgae, and mixed feeders of algae and detritus (Hoey et al. 2013). Croppers and browsers are found in both Siganidae and Acanthuridae, but behavioral feeding comparisons between the families have not been explored at the bite level (but see Streit et al. 2015). Previous work on siganids feeding in situ has found some species to feed on the same tuft of algae for longer periods of time than acanthurids or other herbivorous fishes, a behavior termed “nibbling” (Brandl and Bellwood 2015; Streit et al. 2015). However, this behavior has not been investigated in detail. Thus, it is unclear whether siganids represent a largely parallel family or if they possess unique feeding mechanisms not seen in acanthurids. There is a need, therefore, to identify and understand feeding behavior within Siganidae, as this may provide a functional basis for understanding their ecological niche.

In our study, we conducted comparative analyses of the feeding behavior of rabbitfishes (i.e., siganids) focusing on movements of the jaws and body using laboratory-based high-speed videography of seven species. First, we compared the feeding behavior of siganids to those of the closely related acanthurids. We then carried out a detailed analysis specifically for siganids. With this comparative study, we addressed three main questions; (1) How does feeding behavior in Siganidae differ from Acanthuridae at the bite level? (2) What are the main patterns of diversity in feeding behavior among siganid species? (3) Are there morphological components driving behavioral differences? With these comparisons, we provide a detailed analysis of the different behaviors observed in Siganidae during feeding.

## Materials and methods

### Filming

Individuals of seven species of Siganidae were obtained from commercial vendors, and maintained in the lab in 100- or 200-L tanks, depending on the size of the fish. All individuals were allowed to acclimate for about one week before filming commenced. Once fish were readily feeding on experimental food preparations (see below) experimental periods were initiated. We recorded videos of the following seven species: *Siganus magnificus* (*n* = 3 individuals), *S. unimaculatus* (*n* = 3), *S. vulpinus* (*n* = 3), *S. canaliculatus* (*n* = 3), *S. lineatus* (*n* = 3), S. *stellatus* (*n* = 3), and *S. virgatus* (*n* = 3). Five feeding sequences were analyzed for each individual rabbitfish. The first three species listed possess an elongate snout, whereas the latter four do not. We compared rabbitfish feeding behavior to that of seven species of Acanthuridae using videos from a previous study (Mihalitsis et al. 2025b). These species were: *Prionurus laticlavius* (*n* = 3 individuals, mean sequences per individual = 4), *Acanthurus coeruleus* (*n* = 1, 10 sequences), *Acanthurus chirurgus* (*n* = 2, mean = 6.5 sequences per individual), *Zebrasoma veliferum* (*n* = 3, 5 sequences for each individual), *Acanthurus lineatus* (*n* = 2, mean = 7 sequences per individual), *Acanthurus leucosternon* (*n* = 2, 7 sequences for each individual), and *Acanthurus xanthopterus* (*n* = 2, mean = 7 sequences per individual).

All fish were filmed feeding with either a Fastec-High Spec 2G, or a Photron Fastcam Mini camera, recording at 1000 frames per second. A total of 192 videos were used in our analyses (100 videos of Siganidae and 92 videos of Acanthuridae. The fish were filmed feeding on tufts of filamentous algae which were threaded through holes on a plastic plate that had a grid of holes separated by 5 mm as described previously (Mihalitsis and Wainwright 2024). This setup resulted in the fishes targeting individual tufts of algae. The plastic plate was held in place with elastic bands on a 20 mm thick acrylic block. A ruler was filmed above the experimental setup prior to each filming session to allow distance calibrations for subsequent analysis. All filamentous algae were grown in a single tank in the laboratory under light.

Each recorded feeding sequence consisted of one full bite. A feeding bite is initiated by a fish transitioning from being horizontally oriented in the water column, swimming down to the substratum of the tank, followed by the fish opening its mouth and angling its body in a downward position to feed on the benthos (see Supplemental Fig. 1). A bite was defined as complete when the fish disengages with the algae tuft, either with a pulling motion (i.e., lateral head flick, neurocranium ventral contraction) or returning to a horizontal orientation in the water column. If a fish kept its head down and conducted consecutive opening and closings of the jaws on the same tuft of algae without a pulling motion or disengaging in between, this was scored as nibbling. If the fish conducted a pulling motion, this marked the end of a bite (see Supplemental Fig. 1). Feeding videos were saved for subsequent analyses only if the fish appeared to be in full lateral view of the camera. All video recording was conducted in the lab in accordance with the University of California, Davis Institutional Animal Care and Use Committee, protocol numbers 22206 and 23818.

### Data and statistical analysis

Videos of bites were loaded into the software ImageJ and each sequence was scored for the presence or absence of 10 behaviors (see Table 1). In bites where the fish nibbles, the behaviors were scored as present if it was seen in any of the individual opening and closing of the jaws. Exceptions to this were scoring the presence and absence of nibbling and whether the first individual opening and closing of the jaws involved full closure. The 10 behaviors are motions used to support the core biting actions during feeding and detachment of algae and are consistent with previous literature on herbivorous feeding behavior in laboratory settings (Perevolotsky et al. 2020; Mihalitsis and Wainwright 2024; Perevolotsky et al. 2025; Mihalitsis et al. 2025a; Mihalitsis et al. 2025b). We also note that one of the behaviors described herein has also been described in previous literature of in situ observations (Brandl and Bellwood 2015; Streit et al. 2015). The time of each bite was also measured, with the start of a bite corresponding to the fish initially opening its jaws to feed, and the end of a bite corresponding to the fish conducting a pulling motion to detach algae or disengaging with the substrate.

**Table 1:** Descriptions of 10 feeding behaviors scored in videos of fishes feeding on tufts of filamentous algae.

	Feeding behavior	Description
1	Nibbling	Consecutive opening and closing of the jaws on the same tuft of algae during biting, without a pulling motion in between (e.g., lateral head flick) (see Supplemental Video 1).
2	Extra jaw closing	Jaw closure occurring with the lower jaw extending past the upper jaws after the original closing of jaws (see Supplemental Video 2).
3	Open jaws contact substratum	Upper and/or lower jaw(s) contact the substratum while the gape is open.
4	Gape fully closed upon first jaw closure	The lower jaw is fully adducted upon jaw closure. For bites with nibbling, this refers to the initial jaw closure
5	Pelvic ventral contraction	Contraction of the body between the pelvis, postcleithrum, and the base of the anal fin.
6	Head flick	Lateral (i.e., sideways) head movement during algal detachment.
7	Suction	Movement of algae towards the open mouth of the fish as it engulfs a tuft of algae.
8	Neurocranium ventral contraction	Ventral rotation of the neurocranium during the pulling motion on the algae.
9	Upper jaw lateral motion	Lateral (i.e., sideways) movement of the upper jaw relative to the rest of the head.
10	Lower jaw dorsoventral rotation	Dorsoventral rotation of the lower jaw while the mouth is closed during the pulling motion on the algae.

**Table 2:** Frequency of 10 feeding behaviors for each species analyzed. Numbers represent averages across individuals within a species.

Siganidae	Nibbling	Extra jaw closure	Open jaws contact substrate	Gape fully closed upon first jaw closure	Pelvic ventral contraction	Head flick	Suction	Neurocranium ventral contraction	Upper jaw lateral motion	Lower jaw dorsoventral rotation
*S. stellatus*	93%	7%	100%	7%	33%	67%	87%	0%	0%	0%
*S. virgatus*	93%	93%	93%	60%	27%	67%	47%	0%	0%	0%
*S. lineatus*	100%	73%	33%	80%	33%	80%	27%	0%	0%	0%
*S. canaliculatus*	100%	87%	33%	80%	7%	7%	20%	0%	0%	0%
*S. vulpinus*	73%	47%	0%	100%	13%	13%	93%	0%	0%	0%
*S. magnificus*	30%	0%	60%	80%	10%	10%	50%	0%	0%	0%
*S. unimaculatus*	50%	7%	33%	87%	40%	40%	53%	0%	0%	0%
**Acanthuridae**										
*A. chirurgus*	0%	0%	54%	100%	85%	100%	77%	85%	92%	15%
*A. coeruleus*	0%	0%	90%	100%	90%	70%	90%	100%	50%	20%
*P. laticlavius*	0%	0%	33%	100%	92%	92%	17%	100%	75%	100%
*Z. veliferum*	0%	0%	27%	100%	100%	100%	67%	80%	100%	0%
*A. leucosternon*	0%	0%	7%	100%	100%	72%	36%	100%	0%	100%
*A. lineatus*	0%	0%	0%	100%	79%	93%	93%	86%	21%	79%
*A. xanthopterus*	0%	0%	57%	100%	43%	100%	93%	43%	100%	0%

A Principal Component Analyses (PCA) was performed on the correlation matrix derived from the overall data matrix of presence or absence of the 10 behaviors within bites. We used a PERMANOVA (Permutational Multivariate Analysis of Variance) on the original binary data set of presence and absence of behaviors to test for differences in the overall feeding profile between families and between species. A second PCA was run on the correlation matrix derived from binary presence/absence behavioral data from siganids only, including only the seven behaviors shown by this family. We used a PERMANOVA on this binary data set to compare feeding behavior between long and short-snouted rabbitfish species. To assess whether the frequency of behaviors differed among siganid species, we conducted a Kruskal–Wallis test and a Dunn post-hoc test with behavior as the independent variable and the frequency as the dependent variable. The same approach was repeated for the duration of bites for each species, and the number of individual opening and closings of the jaws when biting (i.e., nibbling). All statistical analyses were performed using the software R.

## Results

### Siganidae versus Acanthuridae feeding behaviors

Of the 10 feeding behaviors scored, five were shared between Siganidae and Acanthuridae. These were: open jaws contacting the substrate, gape fully closed upon first jaw closure, pelvic ventral contraction, lateral head flick, and suction (Table 1). Three behaviors were only found in acanthurids: neurocranium ventral contraction, upper jaw lateral motion, and lower jaw dorsoventral rotation. Furthermore, species within Acanthuridae were observed always fully closing their jaws around tufts of algae, and only conducting a single opening and closing of the jaws before detaching algae from the substrate. Two behaviors were only found in siganids: nibbling and extra jaw closure (Table 1). The maximum number of nibbles we observed within one bite was 22 (*S. canaliculatus)* but nibbling was seen in all siganid species during at least 30% of bites within a species (see Supplemental Video 1).

A PCA and PERMANOVA of behaviors from 192 feeding bites, from 14 species (Table 2) showed substantial and significant differences in feeding behavior among Siganidae and Acanthuridae (*F* = 144.12, *P* < 0.001, Fig. 1). The first two axes of the PCA explained a total of 52.87% of the variance (PC1 = 38.45%, PC2 = 14.42%). The first PC was primarily driven by differences in how siganids and acanthurids detach algae from the substrate. PC1 was negatively correlated with extra jaw closure and nibbling (Fig. 1; see Supplemental Table 1), behaviors routinely performed by siganids but never by acanthurids. PC1 was positively correlated with neurocranium ventral contraction, upper jaw lateral movement, and lower jaw dorsoventral rotation, traits that are characteristic of surgeonfish feeding behavior but never seen in siganids. PC2 was positively correlated with suction, open jaws contacting the substrate, and a lateral head flick, showing variation within both families for these behaviors. A subsequent PERMANOVA at the species-level found significant differences between all siganid species and all acanthurid species. Overall, these results show that the two major groups of cropping herbivorous reef fishes use distinct combinations of behaviors when feeding on tufts of algae.

**Fig. 1:**
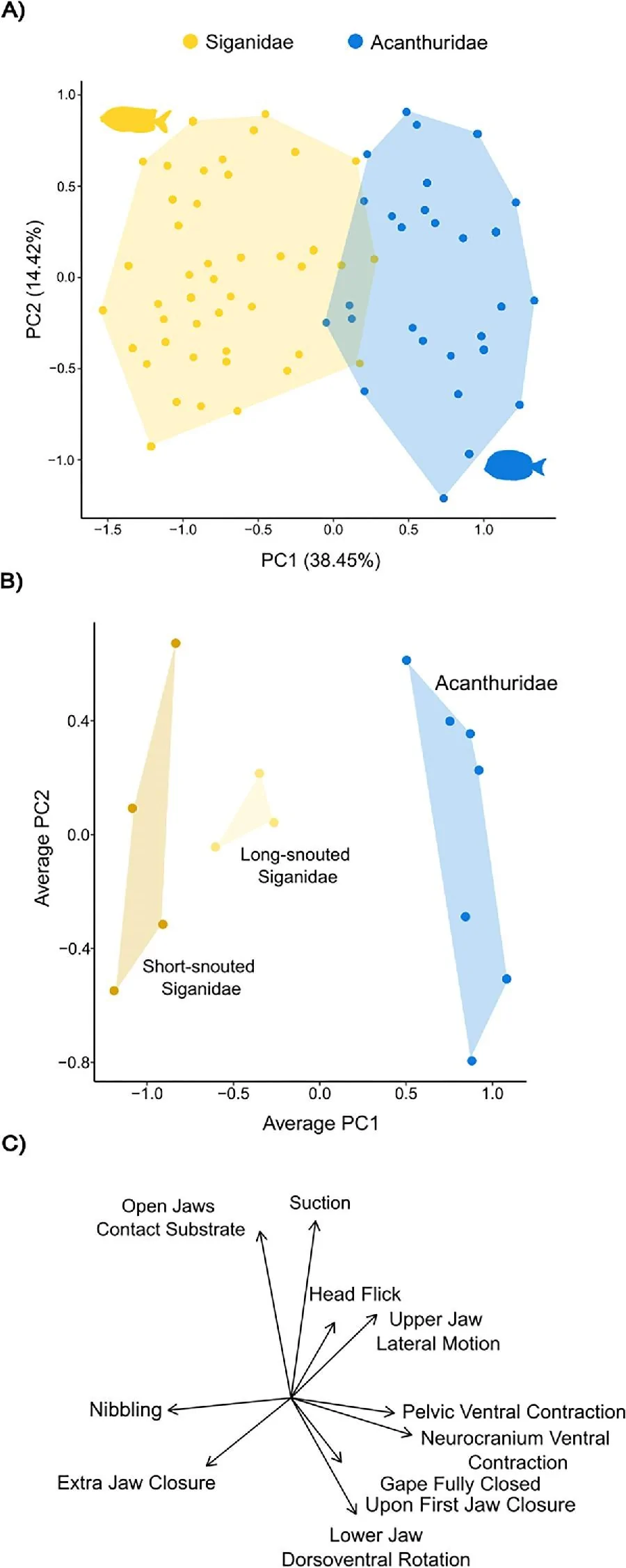
Feeding behaviors across bites by Siganidae and Acanthuridae. (**A**) PCA of behaviors scored. Each point corresponds to a bite. Bites from Siganidae species are shown in yellow and Acanthuridae in blue. (**B**) Average PCA scores by species. Short-snouted Siganidae are shown in dark yellow, long-snouted Siganidae are shown in light yellow, and Acanthuridae are shown in blue. Siganid species in decreasing order of their PC2 score: *S. stellatus, S. magnificus, S. unimaculatus, S. vulpinus, S. virgatus, S. lineatus, S. canaliculatus.* Acanthurid species in order of decreasing PC2 score: *A. xanthopterus, A. coeruleus, A. chirurgus, Z. veliferum, A. lineatus, P. laticlavius, A. leucosternon.* (**C**) Loading vectors from the PCA shown in (A) and (B).

### Rabbitfish-specific feeding behaviors

The PCA and PERMANOVA conducted for only siganid species showed significant separation between bites from short-snouted and long-snouted species (*F* = 38.50; *P* < 0.001; Fig. 2). The first two PC axes explained 51.6% of the total variance (PC1 = 26.91%, PC2 = 24.69%) (Fig. 2). PC1 was negatively correlated with extra jaw closure and nibbling (see supplemental Table 2), behaviors that were more often observed in short-snouted species (Fig. 2). PC1 was positively correlated with suction and conducting a lateral head flick, behaviors more commonly observed in long-snouted species. Positive scores on PC2 are correlated with the gape being fully closing upon the first jaw closure. Negative scores are correlated with open jaws contacting the substratum and pelvic ventral contraction. These results reveal differences in feeding behavior between short-snouted and long-snouted rabbitfishes, mainly along PC1.

**Fig. 2:**
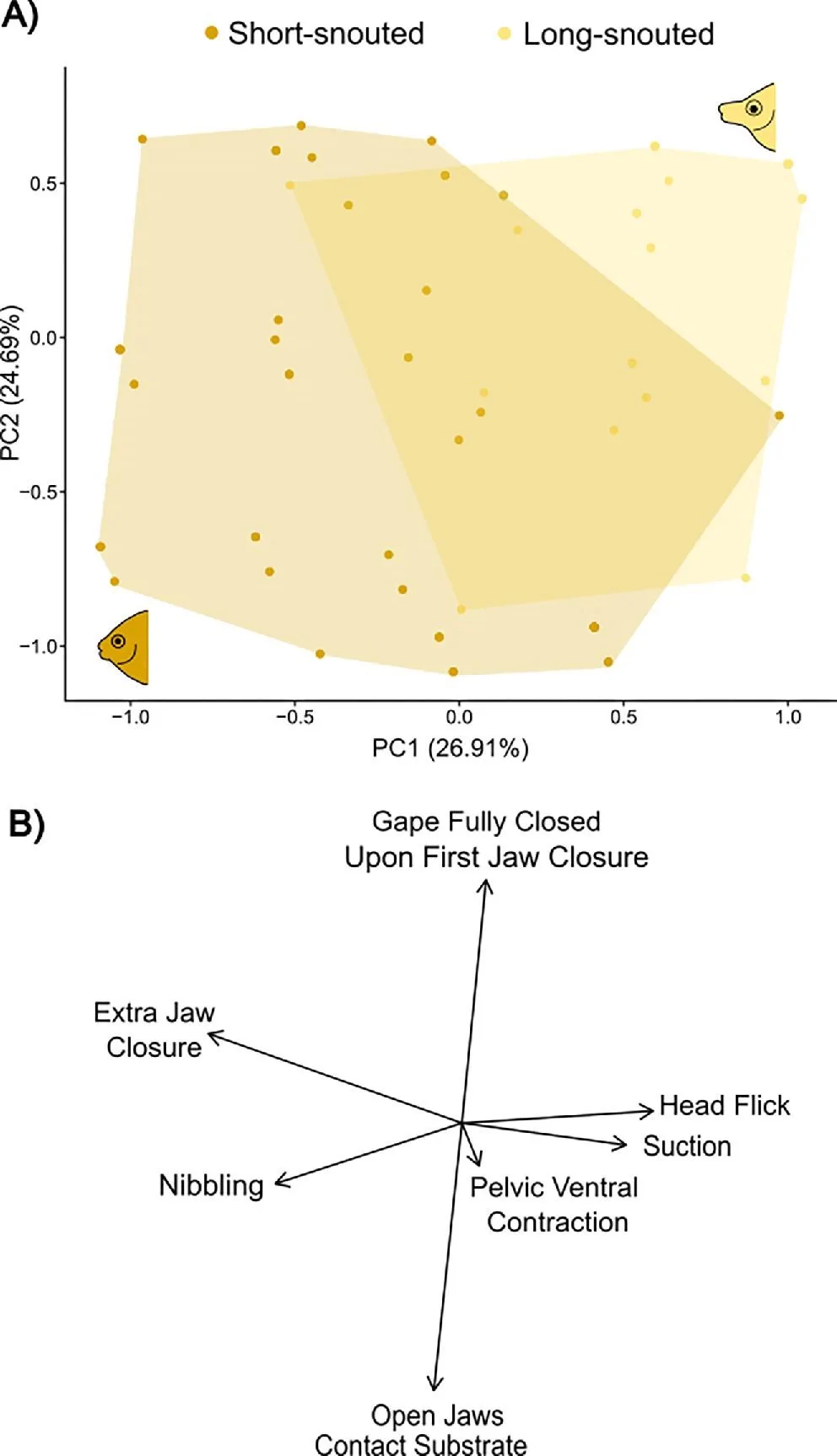
Feeding behaviors within rabbitfishes (Siganidae). (**A**) PCA of behaviors. Each point corresponds to a single bite. Short-snouted species are shown in dark yellow points, and long-snouted species are shown in light yellow. (**B**) Loading vectors from the PCA shown in (A).

Within Siganidae, the same seven feeding behaviors were utilized across all species, however, there were differences in the frequency of usage (Fig. 3). All siganid species were observed to nibble, yet the species-specific frequency ranges from 30 to 100% of bites. Similarly, extra jaw closure ranges from 0 to 93%, suggesting a wide range of species-specific variation in feeding behavior within siganids. The behavior with highest mean frequency across siganid species was the presence of nibbling (mean freq. = 0.77, s.e. = 10, Fig. 4), whereas the least frequent behavior was pelvic ventral contraction. A Kruskal–Wallis test and Dunn post-hoc test identified pelvic contraction (mean freq. = 0.23, s.e. = 0.05) as being significantly less frequent compared to the rest of the behaviors. The other two most common behaviors include a lateral head flick and gape fully closed upon the first jaw closure, both of which are significantly more frequent compared to suction, open jaws contacting the substrate, and extra jaw closure. Out of the seven behaviors observed in siganids, four of these directly involve the jaws: nibbling, gape fully closed upon first jaw closure, extra jaw closure, and open jaws contacting the substrate. These results show the prevalence of jaw movements in siganids as opposed to extensive use of both body-and jaw-related movements in acanthurids.

**Fig. 3:**
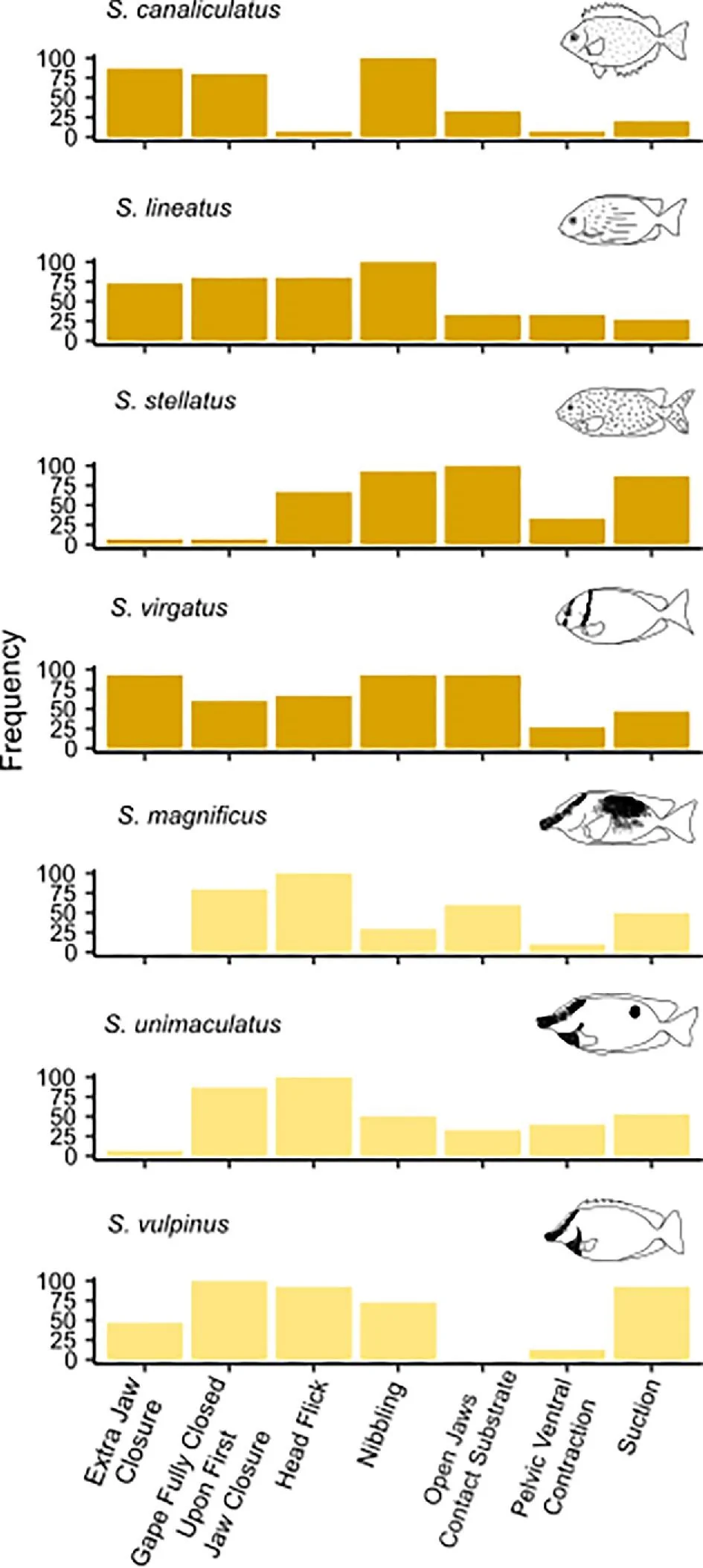
Species-specific frequency of feeding-related behaviors in Siganidae. Each bar corresponds to a behavior (*x*-axis) and the height of each bar represents the frequency of bites the behavior was observed in, for each species. Short-snouted species are shown in dark yellow, whereas long-snouted species are shown in light yellow.

**Fig. 4:**
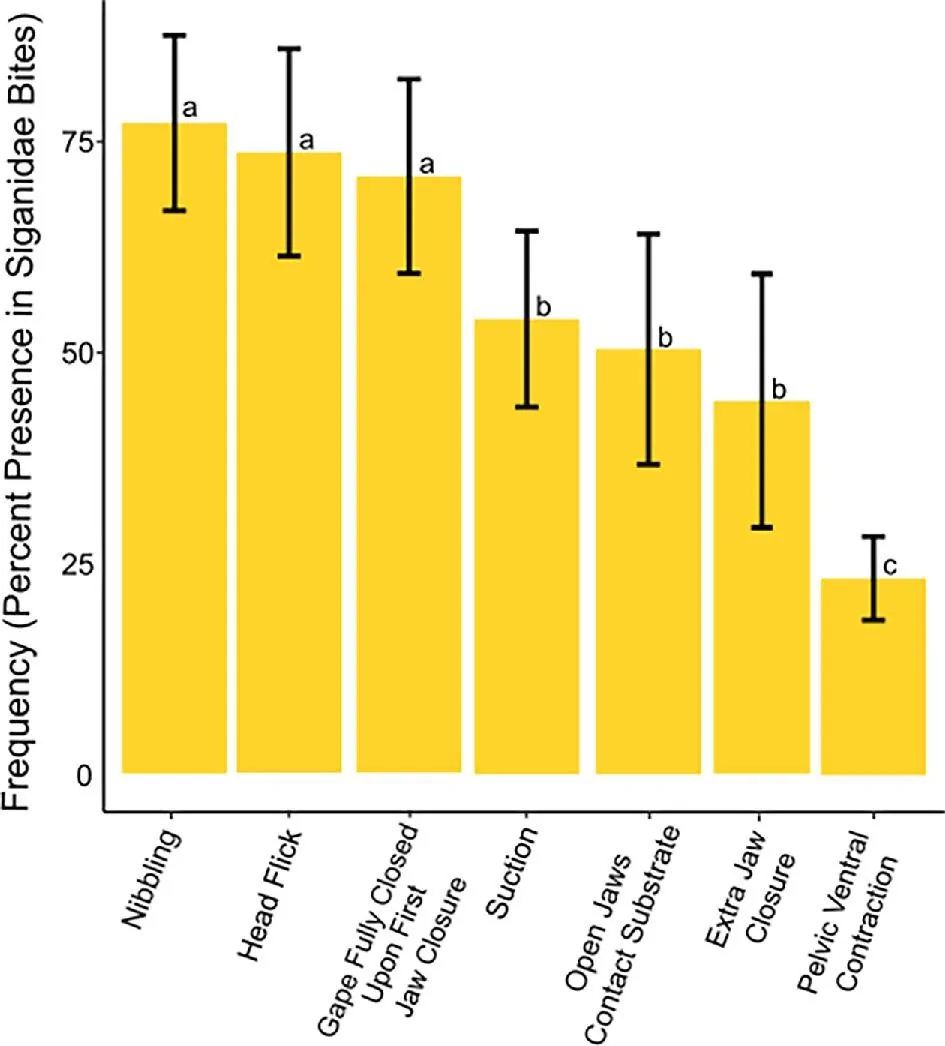
Frequency of feeding-related behaviors in Siganidae. Each bar corresponds to a behavior (*x*-axis). The height of each bar represents the frequency of that behavior across all siganids. Behaviors grouped with the same letter are not significantly different from each other.

The most defining behavior of siganids (Fig. 1), as well as the most prevalent (Figs. 3 and 4), is the presence of nibbling, so we investigated this trait in more detail (Fig. 5). A Kruskal–Wallis test and Dunn post-hoc test identified the schooling and group-forming *S. canaliculatus* and *S. lineatus* as displaying significantly greater numbers of opening and closing of the jaws (i.e., nibbles), compared to pairing species (Fig. 5A). *S. canaliculatus* had a mean number of 10.5 nibbles per bite (s.e. = 1.64), whereas *S. lineatus* had a mean number of 7.3 nibbles per bite (s.e. = 0.91). Taking more nibbles within a bite resulted in a longer bite time (Fig. 5B). *S. canaliculatus* and *S. lineatus* also displayed significantly greater bite times (in milliseconds), compared to the rest of the siganid species (Fig. 5B) (except for *S. virgatus* which was not significantly different from the two species)*. S. canaliculatus* had a mean bite time of 1643 ms (s.e. = 246.7) whereas *S. lineatus* had a mean bite time of 1557 ms (s.e. = 234.8).

**Fig. 5:**
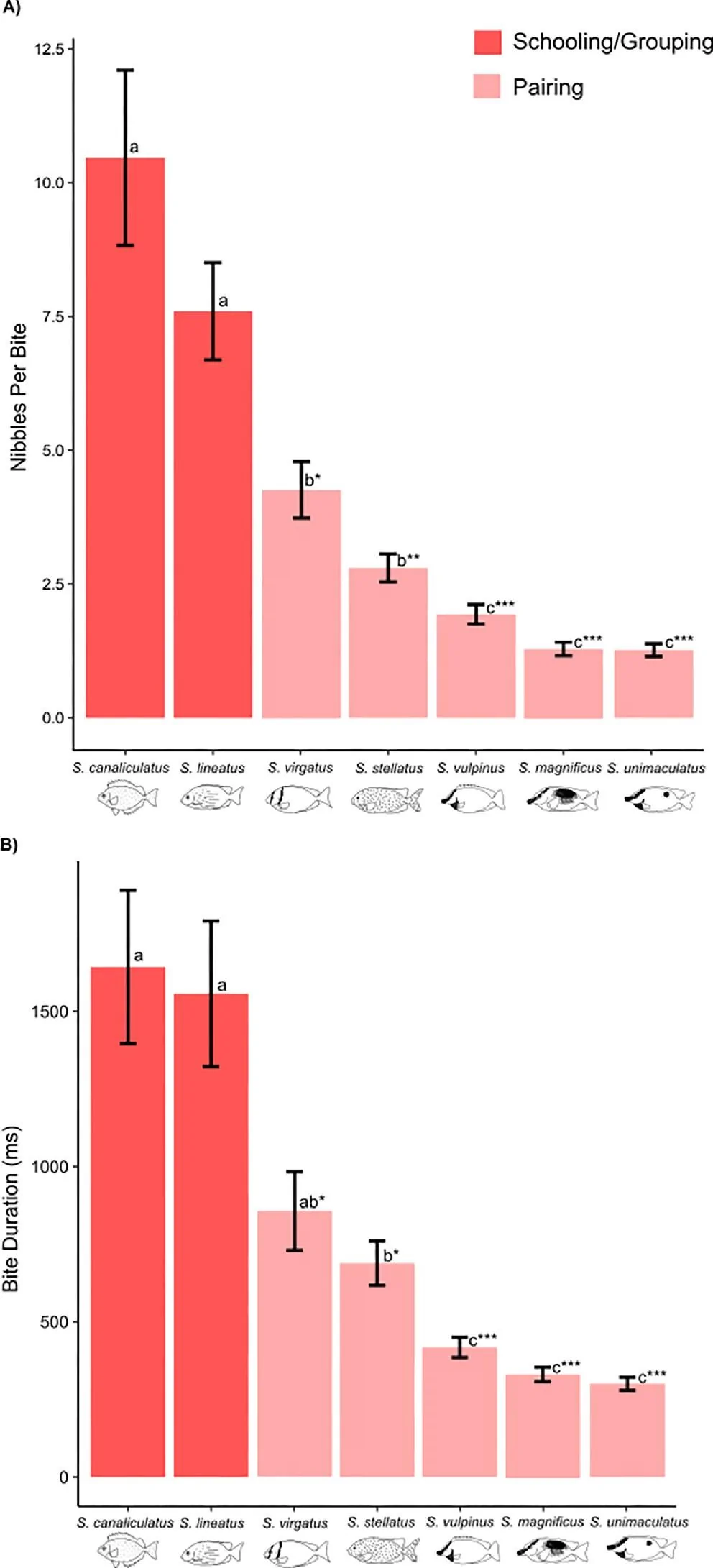
Average number of nibbles and duration of bites in siganid species. (**A**) Average number of nibbles per bite for each rabbitfish species. Schooling and grouping species are shown in dark pink, whereas pairing species are shown in light pink. Species grouped with the same letter are not significantly different from each other. (**B**) Average bite duration in milliseconds for each rabbitfish species.

## Discussion

Our results reveal shared use of a core set of behaviors that are expressed during feeding in siganids and acanthurids, with a set of unique behaviors observed in each group. Although the two groups are both primarily categorized as cropping herbivores, our observations suggest that their methods of detaching algae show differences. These results may thus have important implications to the feeding dynamics of these species on reefs. Siganids show differences in their feeding repertoire along axes of sociality (schooling/grouping vs. pairing) and morphology (short-snouted vs. long-snouted), as well as variation within each species. Overall, our results show that major lineages of herbivorous reef fishes display large behavioral variation directly associated with the detachment of algae, suggesting that algal—herbivore dynamics may be more dynamic and context dependent than previously thought.

### Siganidae versus Acanthuridae feeding

Feeding by siganids involves the use of several behaviors commonly used by acanthurids, plus two behaviors unique to the family (nibbling and extra jaw closure). Similarly, acanthurids often use behaviors not found in siganids. Although siganids and many acanthurids are mostly classified as croppers and are somewhat closely related among acanthomorph fishes (Near and Thacker 2024), we found some differences in how they feed. A key difference in feeding behavior is the presence of nibbling within siganids. We define nibbling as consecutive opening and closing of the jaws during biting, without a pulling motion on the algae or disengaging with the substrate in between cycles of opening and closing. This behavior is unique to siganids when compared to acanthurids. Based on our results, we provide further information on this behavior which was previously only described from in situ studies through diver observations, thus not allowing detailed analysis. We note that these consecutive opening and closing cycles of the jaws appear to be different from the initial jaw opening and closing, with the jaws not opening and closing fully. Furthermore, we note that nibbling appears to be occurring on the same tuft of algae, whereas acanthurids will always take a single bite on an individual algal tuft.

Acanthurids also made use of several movements that support the ability to move their closed jaws relative to the rest of the skull or the body. These include pelvic ventral contraction, neurocranial ventral contraction, ventral flexion of the mandible during the bite, and lateral flexion of the upper jaw. Of these, only pelvic ventral contraction was observed in siganids, although it was much less common when compared to the frequency observed in acanthurids (Fig. 4). We also note that, while not quantified herein, the range of motion during ventral contraction in siganids appeared to be considerably less when compared to acanthurids. This reduced frequency and extent of this behavior is likely related to the modified postcleithrum within siganids that, unlike acanthurids, extends posteriorly and is attached at the base of the first anal-fin pterygiophore (Horn 1989; Tyler and Bannikov 1997). We hypothesize that the post cleithrum bone acts as a body stiffening strut in siganids and thus, restricts contraction in this area, while more motion is possible in acanthurids (Mihalitsis and Wainwright 2024; Mihalitsis et al. 2025b). This restriction in the pelvic region in siganids suggests it is not possible for them to produce the same type of pelvic contraction as acanthurids.

Another morphological feature that varies between the two families, which may explain differences observed between the two families, is that siganids show separate ossifications of the palatine bone, and thus also having a “prepalatine” (Starks 1907; Tyler and Bannikov 1997). This unique morphological feature appears to be derived in siganids, as acanthurids and zanclids have a single ossification, connecting the palatine to the neurocranium (Tyler and Bannikov 1997). While the function of two separate ossifications of the palatine and prepalatine is currently unknown, it is generally accepted that bone ossification occurs at early life stages in vertebrates. Therefore, it suggests that the palatine in larval or juvenile siganids may not be ossified initially. The primary functions of the palatine are to connect the suspensorium to the neurocranium and stabilize rotation of the head of the maxilla and thus aid in a range of jaw motions including jaw protrusion (Alexander 1967). Within acanthurids the palatine plays a role in the innovation of lateral jaw rotation (Mihalitsis et al. 2025a). Therefore, an unossified palatine at an early juvenile stage may have implications related to feeding at that stage. Indeed, previous work has found strong ontogenetic gradients and shifts of both habitat and diet, as well as upper jaw morphology between juvenile and adult siganids (Martin et al. 2024; Nuruki et al. 2026). Further work on the potential function of an unossified two-part palatine in siganids, may thus reveal uncovered links to feeding mechanics, which may in turn, be linked to ontogenetic habitat and diet shifts.

While it is known that algal turfs on reefs can vary substantially in length, composition, and sediment content, our results may also have implications for understanding the functional complementarity of cropping herbivorous reef fishes. Not all croppers feed using the same bite-specific behaviors, supporting previous work on limited functional overlap of herbivorous reef fishes (Burkepile and Hay 2008; Kelly et al. 2016; Streit et al. 2019). Here, we provide new insights into the complementarity of reef fishes through high-speed filming, which reveal that intricacies of cropping may manifest at the bite-specific level, rather than only between species. We show that two closely related groups of fishes have evolved different mechanisms to feed on algae. The use of nibbling was seen in all seven species of siganids but never in any species of acanthurid. This suggests that siganids may remove material completely from a biting location while acanthurids may crop the top layer of turf and move on, or that siganids may be targeting different benthic components than acanthurids.

### Rabbitfish feeding ecology

We find that siganids conduct a nibbling behavior and have the capability to produce a further closing of the jaws past the point where the upper and lower jaw teeth meet, producing an overbite (i.e., extra jaw closure, Table 1). However, the frequency of these behaviors varies by species. We observed S. *canaliculatus* to have the greatest average number of nibbles, supporting field observations of S. *canaliculatus* having a high bite rite when compared to other herbivorous species (Bennett and Bellwood 2011). S. *canaliculatus* is the only species in our study that is classified as a browser, due to its habit of primarily feeding on leathery brown macroalgae (Hoey et al. 2013). This differs from the diet of croppers, which feed mostly on smaller red and green algae (Hoey et al. 2013). This behavioral difference may be due to thicker leathery macroalgae requiring more bites to be cut or detached, compared to other smaller and finer types of algae.

It is known that social behavior has implications on biting dynamics of benthic feeding herbivorous reef fishes (Robertson et al. 1976; Reinthal and Lewis 1986; Wolf 1987; Michael et al. 2013). Conducting a greater number of nibbles per bite leads to more time for a fish to have its head down in the substrate, and less time keeping a watch for predators. In previous studies with several species, individuals in groups were found to have higher bite rates than solitary fishes, an effect attributed to a decreased risk of predation when schooling (Green and Côté 2014; Basford et al. 2015; Reinthal and Lewis 1986). Based on previous literature, *S. canaliculatus* forms small schools, *S. lineatus* forms small groups, and the rest of the species form pairs (Brandl and Tebbett 2025). We observe schooling and grouping species to have an average bite time of 1600 ms (s.e. = 240.8) whereas pairing species have an average bite time of 519.4 ms (s.e. = 55.04) (Fig. 5D). In other words, siganids that form small groups and schools spend up to three times longer with their head down in the substrate when feeding than pairing siganids. Thus, our results add another component, time spent feeding within a bite, to the already established relationship between sociality and feeding behavior within siganids. We note that morphology, diet, and sociality within rabbitfishes have previously been acknowledged as intertwined (Brandl and Tebbett 2025). While our study was not able to separate the independent effects of sociality and snout morphology, our results show that these previously suggested ecological drivers persist, even at the bite-specific level.

## Conclusion

We found key differences between siganids and acanthurids in the behaviors that accompany basic biting actions used when feeding. These families share a core set of motions, yet also exhibit family-specific behaviors. Species within each family vary in the frequency of use of behaviors found in each group. We suggest that future work expands on our results by identifying potential performance trade-offs and how trophic morphology and behavior shape the feeding abilities of species in these two groups.

## Supplementary Material

obag056_Supplemental_Files

## Data Availability

The data underlying this article are available in Figshare, at https://figshare.com/projects/Functional_distinctions_in_feeding_behavior_of_two_herbivorous_reef_fish_lineages/275940
