# Supplementary material for: Functional Distinctions in Feeding Behavior of Two Herbivorous Reef Fish Lineages
Source: Integr Org Biol. 2026 Sep 21;8(1):obag056. doi: 10.1093/iob/obag056 (PMC13714546; doi:10.1093/iob/obag056)
Supplement: Supplementary file 1 [file obag056_supplemental_files.zip › Supplemental Table 1.docx]

**Supplemental Table 1:** Loadings for the first two PC axes from a PCA on behavioral data from all videos.

| **Loading Vector** | **PC1** | **PC2** |
| --- | --- | --- |
| Nibbling | -0.48 | -0.04 |
| Extra jaw closure | -0.33 | -0.22 |
| Open jaws contact substrate | -0.12 | 0.54 |
| Gape fully closed upon first jaw closure | 0.20 | -0.21 |
| Pelvic ventral contraction | 0.40 | -0.05 |
| Head flick | 0.17 | 0.24 |
| Suction | 0.01 | 0.57 |
| Neurocranium ventral contraction | 0.47 | -0.12 |
| Upper jaw lateral motion | 0.34 | 0.27 |
| Lower jaw dorsoventral rotation | 0.25 | -0.38 |
