# Supplementary material for: Functional Distinctions in Feeding Behavior of Two Herbivorous Reef Fish Lineages
Source: Integr Org Biol. 2026 Sep 21;8(1):obag056. doi: 10.1093/iob/obag056 (PMC13714546; doi:10.1093/iob/obag056)
Supplement: Supplementary file 1 [file obag056_supplemental_files.zip › Supplemental Table 2.docx]

**Supplemental Table 2:** Loadings for the first two PC axes from a PCA on behavioral data from videos of Siganidae.

| **Loading Vector** | **PC1** | **PC2** |
| --- | --- | --- |
| Nibbling | -0.46 | 0.16 |
| Extra jaw closure | -0.63 | 0.23 |
| Open jaws contact substrate | -0.07 | -0.70 |
| Gape fully closed upon first jaw closure | 0.06 | 0.64 |
| Pelvic ventral contraction | 0.04 | -0.11 |
| Head flick | 0.47 | 0.03 |
| Suction | 0.40 | -0.06 |
